# Pancreatic and duodenal homeobox 1 (PDX1) phosphorylation at serine-269 is HIPK2-dependent and affects PDX1 subnuclear localization

**DOI:** 10.1016/j.bbrc.2010.07.035

**Published:** 2010-08-20

**Authors:** Rong An, Gabriela da Silva Xavier, Francesca Semplici, Saharnaz Vakhshouri, Huai-Xiang Hao, Jared Rutter, Mario A. Pagano, Flavio Meggio, Lorenzo A. Pinna, Guy A. Rutter

**Affiliations:** a Section of Cell Biology, Division of Diabetes, Endocrinology and Metabolism, Department of Medicine, Imperial College London, London SW7 2AZ, UK; b Department of Biochemistry, University of Utah, Salt Lake City, UT 84112-5650, USA; c Department of Biological Chemistry, University of Padova, viale G. Colombo 3, 35131 Padova, Italy

**Keywords:** PDX1, Islet, β-Cell, Phosphorylation, Glucose, HIPK2

## Abstract

Pancreatic and duodenal homeobox 1 (PDX1) regulates pancreatic development and mature β-cell function. We demonstrate by mass spectrometry that serine residue at position 269 in the C-terminal domain of PDX1 is phosphorylated in β-cells. Besides we show that the degree of phosphorylation, assessed with a phospho-Ser-269-specific antibody, is decreased by elevated glucose concentrations in both MIN6 β-cells and primary mouse pancreatic islets. Homeodomain interacting protein kinase 2 (HIPK2) phosphorylates PDX1 *in vitro*; phosphate incorporation substantially decreases in PDX1 S269A mutant. Silencing of HIPK2 led to a 51 ± 0.2% decrease in Ser-269 phosphorylation in MIN6 β-cells. Mutation of Ser-269 to phosphomimetic residue glutamic acid (S269E) or de-phosphomimetic residue alanine (S269A) exerted no effect on PDX1 half-life. Instead, PDX1 S269E mutant displayed abnormal changes in subnuclear localization in response to high glucose. Our results suggest that HIPK2-mediated phosphorylation of PDX1 at Ser-269 might be a regulatory mechanism connecting signals generated by changes in extracellular glucose concentration to downstream effectors via changes in subnuclear localization of PDX1, thereby influencing islet cell differentiation and function.

## Research highlights

► Mouse PDX1 Ser-269 is phosphorylated in pancreatic islets of Langerhans and beta cells. ► High glucose concentration decreases the degree of phosphorylation on PDX1 Ser-269, as assessed by a phospho-specific anti-phospho-Ser269 antibody. ► HIPK2 is a potential kinase for PDX1 Ser-269. ► Phosphorylation at Ser-269 affects the subnuclear distribution of PDX1.

## 1 Introduction

Control by glucose of preproinsulin gene transcription in pancreatic islet β-cells involves the interplay of multiple *trans*- and *cis*-activating factors ([1] and references therein). Pancreatic and duodenal homeobox 1 (PDX1) was first described as a preproinsulin promoter A3-site binding factor [2,3]. PDX1 is involved in both the development and function of pancreatic β and other islet cells [4].

Deletion or inactivation of the *PDX1* gene in the mouse [5] or in man [6] results in the complete failure of normal pancreatic development. Moreover, β-cell selective disruption of murine PDX1 expression [7] or function [8] lead respectively to defective insulin secretion and glucose signaling. On the other hand, heterozygosity for a defective *PDX1* gene leads to defective preproinsulin gene expression [7] and abnormal insulin secretion [9] in mice and to maturity-onset-diabetes of the young 4 (MODY4) in man [6].

In contrast to the relatively well-studied functions of the N-terminus and the homeodomain of PDX1, the role of the conserved C-terminus is less well defined. Mutations which affect the C-terminus of PDX1 are associated with the development of type 2 diabetes in humans [10–12], while other findings indicate that the C-terminal domain may serve as both repressor and activator of PDX1 function [13,14].

Humphrey and colleagues [15] reported that PDX1 phosphorylation in primary rat islets is decreased by high glucose levels. These authors described Ser-268 and Ser-272 of rat PDX1 (corresponding to Ser-269 and Ser-273 of mouse PDX1) as a novel C-terminal atypical non-primed GSK-3 consensus site which regulates PDX1 protein stability in response to glucose. Importantly, homeodomain interacting protein kinase 2 (HIPK2) ([16] and references therein) has been shown to co-localize with PDX1 in both the developing and adult pancreas and to modulate positively PDX1 transcriptional activity, possibly by phosphorylation of the C-terminal domain [17]. We have previously observed that, in clonal β-cells, elevated glucose concentrations lead to translocation of PDX1 between the nuclear periphery and the nucleoplasm, accompanied by increased preproinsulin promoter activity [18]. Although the molecular basis for the enhanced nucleoplasmic accumulation of PDX1 is unclear, this process may involve interaction of PDX1 homeodomain with the nuclear import receptor family member importin-β1 [19]. In the present study we used mass spectrometry and generated an anti-phospho-serine-specific antibody to confirm Ser-269 as a *bona fide* phosphorylation site in mouse PDX1 that is regulated by glucose in MIN6 β-cells and in primary mouse islets of Langerhans. We show that Ser-269 is phosphorylated by homeodomain interacting protein kinase 2 (HIPK2) *in vitro.* The analysis of (de)phospho-Ser-269-specific mutants suggest that phosphorylation at this site, whilst having no effect on PDX1 protein stability or PDX1 DNA-binding property, is involved in nucleoplasmic (versus nuclear-peripheric) localization in the β-cell in response to glucose.

## 2 Materials and methods

The work described in this article has been carried out in accordance with the *EC Directive 86/609/EEC for animal experiments* http://europa.eu.int/scadplus/leg/en/s23000.htm; and the *Uniform Requirements for manuscripts submitted to Biomedical journals* http://www.nejm.org/general/text/requirements/1.htm.

### 2.1 Cell culture and reagents

Human embryonic kidney (HEK) 293 cells were cultured in Dulbecco’s modified Eagle’s medium (DMEM) (Lonza) containing 10% (v/v) fetal bovine serum (FBS), 100 IU/ml penicillin and 100 IU/ml streptomycin. INS-1(832/13) cells (kindly provided by Dr. C. Newgard, Duke University) and MIN6 β (mouse insulinoma pancreatic beta) cells were cultured as in [20].

Anti-*c-myc* antibody was from Roche. Rabbit polyclonal anti-PDX1 antibody was as described [18]. Anti-phospho-Ser-269-PDX1 antibody was raised in rabbits by immunization with synthetic phospho-peptide: L^262^PSGLSV**pS**PQPSSIAPLRPQEPR^284^ (Pacific Immunology Inc, USA). HIPK2 was purchased from Upstate (Lake Placid, NY).

### 2.2 Mouse islet isolation and culture

Islets were isolated from CD1 mice and cultured as previously described [21].

### 2.3 Plasmids

Plasmid pcDNA3-PDX1-*c-myc* has been described [18]. Mutant plasmids pcDNA3-PDX1-S269A*-c*-*myc* and pcDNA3-PDX1-S269E*-c-myc* were generated using a QuikChange site-directed mutagenesis kit (Stratagene). Wild-type and mutant PDX1 myc-tagged coding sequences were inserted (*Hind*III/*Eco*RV) into the shuttle vector pAdTrack-CMV multiple cloning site [22]. Mouse wild-type PDX1 sequence PCR amplified from plasmid pcDNA3-PDX1-*c-myc* was cloned (*Nco*I/*Bam*HI) into 6xHis-MBP (maltose binding protein) plasmid. Mutant plasmid His-MBP-PDX1 (S269A) was generated as above.

### 2.4 His-MBP-PDX1 production and purification

His-MBP-PDX1 proteins expression was induced in *Escherichia coli* BL21 with 0.2 mM isopropyl-β-d-thiogalactopyranoside (IPTG). Proteins were purified on a nickel–nitrilotriacetic acid column according to Qiagen and dialyzed for 16 h at 4 °C in 50 mM Tris pH 7.9, 150 mM NaCl, 5 mM MgCl_2_, 1 mM β-mercaptoethanol. The MBP moiety was cut with Tobacco Etch Virus (TEV) protease, AcTEV^TM^ protease (Invitrogen). MBP, histidine tag and histidine-tagged Ac-TEV protease were removed respectively with Amylose beads (New England BioLab) and Ni–NTA agarose beads.

### 2.5 Recombinant adenoviruses and viral infection

Recombinant adenoviruses expressing wild-type (WT) and mutant (S269A, S269E) PDX1 and control adenovirus, expressing green fluorescent protein (Ad-GFP) were prepared using the AdEasy system [22]. Cells were infected with various adenoviruses at a multiplicity of infection (MOI) of 50 for 5 h and maintained in 25 mM glucose for 24 h before subsequent experiments.

### 2.6 Real-time RT-PCR

Total mRNA and real-time quantitative RT-PCR analysis was as [23]. Primer sequences are as follows: cyclophilin A fwd, 5′-TAT CTG CAC TGC CAA GAC TGA-3′; cyclophilin A rev, 5′-CCA CAA TGC TCA TGC CTT CTT TCA-3′; HIPK2 fwd, 5′-TGC TTG ACT TCC CCC ATA GTG -3′; HIPK2 rev, 5′-CTT GCA AAT CTC CAT GTT TTG G -3′.

Data were analyzed by ABR PRISM SDS v1.3.1 (Applied Biosystems).

### 2.7 Immunocytochemistry

MIN6 β-cells infected with wild-type or mutant forms of PDX1 viruses at 50 MOI (multiplicity of infection). Immunocytochemical analysis was performed as described in the figure legends and in [20].

### 2.8 Immunoprecipitation

Cells lyzed on ice with 750 μl of IP buffer [1% (v/v) NP40 (Nonidet P40); 50 mM NaCl; 1% (w/v) sodium deoxycholate; 0.1% (w/v) sodium monododecyl sulfate (SDS); 50 mM Tris–HCl pH 7.5; 2 mM EDTA; 10 mM sodium phosphate; 50 mM NaF (sodium fluoride); 1 m PMSF; 200 μM Na_3_VO_4_ (sodium orthovanadate); 1 × Complete^TM^ protease inhibitor cocktail solution (Roche) and 1 × phosphatase inhibitor cocktail 1 & 2 (Sigma)] were rotated on wheel at 4 °C for 30 min. The lysate was clarified by centrifugation at 16,000*g* for 5 min. *c-myc* antibody-conjugated beads (Santa-Cruz; 40 μl) were added and tubes rotated at 4 °C overnight. After centrifugation at 1,000*g* at 4 °C for 30 s, beads were washed five times with lysis buffer.

### 2.9 *In vitro* phosphorylation by HIPK2

Purified wild-type (WT) or serine to alanine mutant (S269A) PDX1 (2 μg) was subjected to *in vitro* phosphorylation with HIPK2 (Upstate) following the manufacturer’s instruction.

### 2.10 Western blotting

Nuclear proteins were extracted as in [24]. Protein samples were separated on SDS–PAGE, and analyzed by Western blotting with the indicated antibodies.

### 2.11 Silencing of HIPK2

siRNA were made with the AMBION Silencer siRNA construction kit. Starting primer pairs were designed as suggested in the AMBION protocol and according to the guidelines published in [25]. Sense and anti-sense primers were derived from mouse cDNA sequence for HIPK2 in the ENSEMBL database (transcript name: Hipk2-201; transcript Id: ENSMUST00000038777). Target sequence was from nucleotide 788 to nucleotide 808 as follows: 5′-AAA CGG GGC ACC AAT GAA ATT CCT GTC TC-3′). Control siRNA were made with primers derived by scrambling the siRNA sequence for HIPK2. All sequences were searched against known mouse cDNA sequences using BLAST (http://blast.ncbi.nlm.nih.gov/Blast.cgi). Transfection was with TransIT-TKO transfection reagent (Mirus Bio Corporation).

### 2.12 Pulse-chase

MIN6 β-cells were infected with PDX1 wild-type, S269A and S269E adenoviruses. 24 h after infection, media were replaced with fresh DMEM containing 3 mM glucose and cells were cultured for 16 h. Cells were transferred for 1 h to cysteine/methionine-free medium, pulsed for 3 h with 0.6 mCi [^35^S]cysteine/methionine (Perkin–Elmer), after which cells were washed and cultured for 2, 4, 8, and 16 h in 5 ml chase media with 10,000-fold molar excess of cold l-cysteine, l-methionine or harvested as the zero time point. Cells were lyzed in 1 ml RIPA [50 mM Tris (pH 7.5), 150 mM NaCl, 1% (v/v) Nonidet P-40 (NP-40) 0.5% (w/v) sodium deoxycholate (DOC), 0.1% (v/v) SDS, 0.4 mg/ml AESBF {4-(2-Aminoethyl) benzenesulfonyl fluoride hydrochloride}, 10 μg/ml Leupeptin, 10 μg/ml Pepstatin, 5 μg/ml Aprotinin, 1 × Complete^TM^ (Roche) and 1 × phosphatase inhibitor cocktail 1 and 2 (Sigma)]. Lysates were immunoprecipitated with anti-myc antibody (Roche). After five washes with RIPA buffer, proteins were eluted and analyzed by SDS–PAGE. PDX1 proteins were detected by fluorography and quantified in a Cyclone (Perkin–Elmer) phosphoimager storage system.

### 2.13 Statistics

Data are given as means ±SE of three to five individual experiments. Comparisons between means were performed using one-tailed Student’s *t*-test for paired data with Bonferroni correction for multiple sampling as appropriate.

## 3 Results

### 3.1 Ser-269 phosphorylation is regulated by glucose in living β-cells (MIN6) and primary mouse islets

**Fig. 1:**
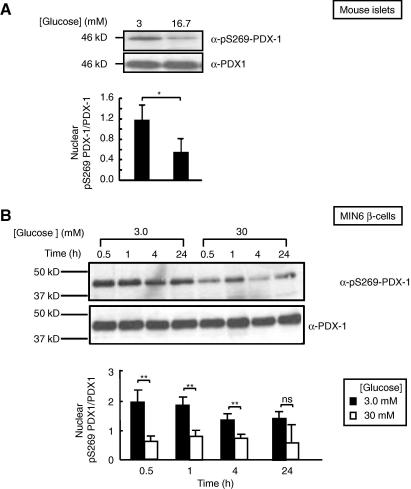
Phosphorylation on Ser-269 is regulated by glucose concentrations in pancreatic islets and MIN6 β-cells. (A) Mouse pancreatic islets of Langerhans or (B) MIN6 β-cells cultured in medium containing 3 mM glucose for 1 and 16 h, respectively. Mouse islets were further cultured for 1 h at three or 16.7 mM glucose. MIN6 β-cells were further cultured at the indicated glucose concentrations for the indicated time. Nuclear lysates were separated on SDS–PAGE gels and analyzed by Western blot using anti-phospho-Ser-269 PDX1 and anti-PDX1 antibodies. The blots shown are representative of 3 independent experiments. The ratio of phospho-Ser-269 PDX1 to total PDX1 level are shown graphically (A, B lower panels). ∗*p* < 0.05, ∗∗*p* < 0.01, ns: non-significant; samples were prepared in triplicate. Mean values from three independent experiments are shown.

PDX1 Ser-269 was identified as a potential phosphorylation site by mass spectrometry (Supplementary Fig. 1). We, therefore, explored the possibility that phosphorylation of PDX1 at Ser-269 may be regulated by glucose in living β-cells and, thus, may, at least potentially, contribute to the regulation of PDX1 function by the sugar. After treatment at low glucose concentrations (3 mM) for 16 (MIN6 β-cells) or 1 h (islets), we further incubated islets (Fig. 1A) or MIN6 β-cells (Fig. 1B) in medium containing 16.7 or 30 mM glucose, respectively, for varying times.

Examined in mouse islets, the nuclear content of phosphorylated Ser-269-PDX1 decreased following culture for 1 h at 16.7 mM glucose (Fig. 1A). In MIN6 β-cells, as early as 30 min after the change in glucose concentration, cells maintained at 30 mM glucose displayed a markedly lower degree of phosphorylation at Ser-269 PDX1 (Fig. 1B). This difference increased, with enhanced phosphorylation at Ser-269, during the subsequent 24 h.

### 3.2 HIPK2 phosphorylation of PDX1

**Fig. 2:**
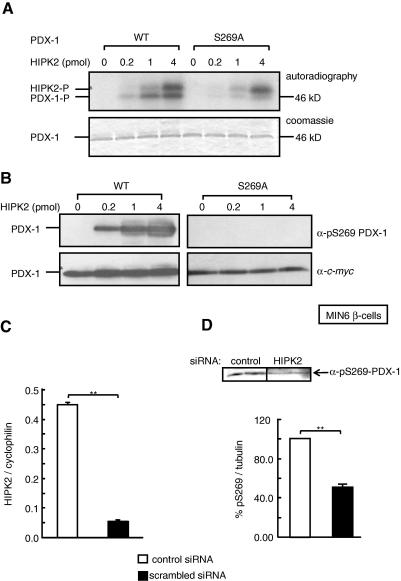
Phosphorylation on Ser-269 by HIPK2 *in vitro* and *in vivo*. (A) 2 μg of recombinant PDX1-*c*-*myc* wild-type or S269A was mixed with (pmol, picomoles) of HIPK2 and [γ-^32^P] ATP for 30 min. Autoradiography shows the radiolabeled PDX1 and HIPK2. Coomassie staining shows the PDX1 protein level. (B) Parallel experiment with cold ATP, analyzed by Western blot using indicated antibodies. MIN6 β-cells were cultured in the presence of 1 nM HIPK2 or scrambled siRNA for 72 h prior to analysis by real-time PCR using primers against mouse HIPK2 (C) or Western blotting with anti-phospho-Ser-269 PDX1 antibody (D). ∗*p* < 0.05, ∗∗*p* < 0.01. Data shown are from a minimum of three independent experiments.

Homeodomain interacting protein kinase 2 (HIPK2) was previously reported to phosphorylate the C-terminal domain of PDX1 at undefined site(s) lying between amino acids 215 and 283 [17]. Since HIPK2 is a proline-directed kinase [17], and Ser-269 is adjacent to a proline residue, we investigated whether Ser-269 may be phosphorylated by HIPK2 *in vitro*. This possibility was examined using active HIPK2 and recombinant *c-myc*-tagged wild-type or S269A PDX1 as substrates (Fig. 2). Wild-type PDX1 incorporated [^32^P] proportionately with increasing concentration of HIPK2, in agreement with the previous study [17] (Fig. 2A). Furthermore, incorporation of [^32^P] into recombinant S269A PDX1 was substantially decreased at all levels of HIPK2 activity tested when compared to the wild-type factor (Fig. 2A). Parallel experiments performed with non-radioactive ATP produced an immunoreactive band at ∼46 kDa with anti-phospho-Ser-269 PDX1 antibody for wild-type PDX1; while, as anticipated, a lack of immunoreactivity was apparent after treatment of S269A PDX1 (Fig. 2B).

Whilst the increased signal detected with the anti-phospho-Ser-269-specific PDX1 antibody in wild-type PDX1 confirmed HIPK2 mediated phosphorylation at this site, residual incorporation of phosphate into S269A PDX1 (Fig. 2A) suggested the existence of additional phosphorylation sites for this kinase. Indeed, our mass spectrometry (Supplementary Fig. 1) identified a long peptide, S^211^SGT^214^PSGGGGGEEPEQDCAVTSGEELLAVPPLPPPGGAVPPGVPAAVR that was also phosphorylated. Although the exact site of phosphorylation on this peptide was not pursued further, we noted a Thr-214 followed by a proline residue in this peptide that meets the criteria for phosphorylation by proline-directed kinases, including HIPK2.

Confirming that Ser-269 phosphorylation occurs via HIPK2 in living cells, MIN6 β-cells in which HIPK2 was silenced by 87.5 ± 1.0% after 72 h culture with siRNA (Fig. 2C), as assessed by real-time PCR analysis (a commercial antibody that recognizes endogenous HIPK2 not being available), had a 51 ± 0.2% decrease in phospho-Ser-269 PDX1 content (Fig. 2D) after 24 h culture at 3 mM glucose.

### 3.3 Phosphorylation on Ser-269 does not affect the stability of PDX1

**Fig. 3:**
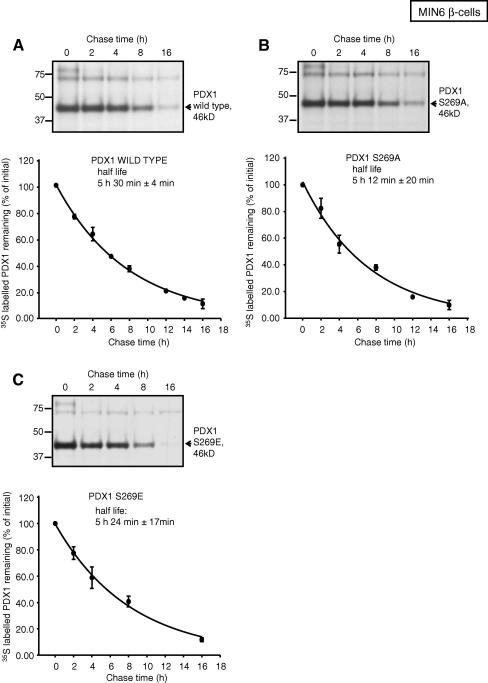
Wild-type and mutant PDX1 half-life MIN6 β-cells were transduced with adenoviruses encoding wild-type *c-myc*-tagged-PDX1 (A) or S269A and S269E mutants (B, C) and their respective protein half-life was measured. Total extracts prepared at the indicated times of chase were immunoprecipitated with anti-*c-myc* antibody. PDX1 protein was detected by fluorography. The intensity of radioactive bands was measured and plotted as a percentage of the initial band intensity (0 h) with Optiquant in a Cyclone (Perkin–Elmer) phospho imager storage system. No significant difference in turnover rate was found between wild-type PDX1 and the mutants. The estimated half-lives of WT PDX1 and mutants S269A, S269E were extrapolated by non linear regression from the exponential decay curve fitted to the data points using SigmaPlot 11.0 (Systat Software Inc., San Jose, CA USA). Data points represent the mean ± SEM of three to four experiments.

We examined the half-life of the mutants by [^35^S] pulse chase labeling experiments which revealed equal stability of phospho/de-phospho-Ser-269 mimetic mutants of PDX1 (Fig. 3). We demonstrated that wild-type *c-myc*-tagged-PDX1 has an estimated half-life, when over expressed in MIN6 β-cells, of 5 h and 30 min (±4). The turnover rates of S269A and S269E PDX1 were not significantly different from wild-type PDX1 (Fig. 3).

### 3.4 Phosphorylation at Ser-269 affects the subnuclear distribution of PDX1 in MIN6 β-cells

**Fig. 4:**
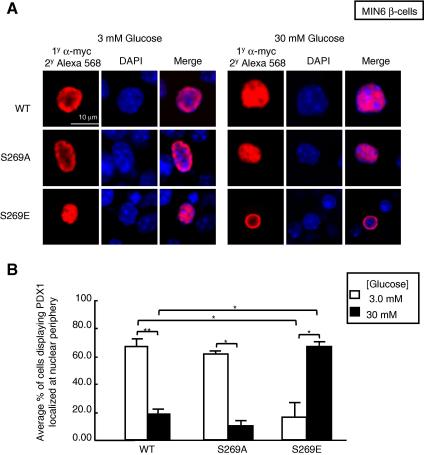
Ser-269 phosphorylation and PDX1 subnuclear localization (A) Immunocytochemical analysis of the subcellular distribution of PDX1-*c-myc*. MIN6 β-cells were transduced with adenoviruses encoding for the indicated PDX1 molecules. Cells were cultured at 3 mM glucose for 16 h and then treated with 3 or 20 mM glucose for 6 h. *c-myc*-tagged PDX1 was detected using anti-*c-myc* antibody (Roche) and Alexa-568 (Molecular Proves). Nuclear staining was achieved using DAPI. Confocal images were captured using a Leica SP2 upright laser scanning confocal microscope (x63/1.32 oil-immersion lens) equipped with a krypton/argon laser (488 and 568 nm excitation lines) and UV light. Images were analyzed off-line using Volocity^TM^ 4.0 software. Each sample was prepared in triplicate. (B) The average number of cells displaying PDX1 localization predominantly at nuclear periphery is expressed as a percentage of the total number of cells analyzed. Mean values from three independent experiments are shown.

In order to determine whether de-phosphorylation at Ser-269 at elevated glucose concentrations may contribute to the nucleoplasmic accumulation of PDX1, MIN6 β-cells were transduced with adenoviruses encoding c-*myc*-tagged wild-type, S269A or S269E mutant forms of PDX1. Cells were then cultured at either 3 or 30 mM glucose. High glucose caused a significant redistribution both of the de-phosphomimetic mutant S269A PDX1 and wild-type PDX1 from nuclear periphery to nucleoplasm (Fig. 4), consistent with previous studies [18]. Thus, at 30 mM glucose wild-type PDX1 was predominantly present in the nuclear region, as was the de-phosphomimetic mutant, while the phospho-mimetic mutant displayed a different localization (nuclear periphery) as compared to wild-type PDX1.

## 4 Discussion

The principal aim of this study was to elucidate the molecular mechanisms involved in the regulation by glucose and other stimuli of the nuclear uptake, DNA binding and transactivation capacities of PDX1, focusing on the potential role of protein phosphorylation.

We demonstrate firstly through mass spectrometry, and by subsequent verification with a phospho-specific antibody, that Ser-269 in the C-terminal domain of PDX1 is phosphorylated in living β-cells, consistent with recent findings [15]. We show here that Ser-269 is de-phosphorylated in response to glucose and that HIPK2 is capable of phosphorylating this residue both *in vitro* and *in vivo*. Phosphorylation of the C-terminus of PDX1 in a region encompassing Ser-269 by HIPK2 has previously been reported, although the effects of glucose on this phenomenon were not described [17]. We show that the degree of phosphorylation of PDX1 at Ser-269 was decreased at elevated glucose concentrations. A detailed analysis of the possible effects of phosphorylation at the site using (de)phosphomimetic mutants failed to reveal any differences between the transactivation capacity of the wild-type protein compared to mutants bearing A or E in place of S269 (Supplementary Fig. 4). These observations, together with the analysis of the subnuclear localization of WT, S269A and S269E PDX1 in MIN6 β-cells (Fig. 4), suggest that HIPK2 phosphorylation of PDX1 regulates PDX1 function via the regulation of its subnuclear localization but not its transactivation potential.

HIPK2 regulates gene expression by phosphorylation of transcription factors (including p53, Pax6, Myb) and accessory components of the transcription machinery (among others p300, CtBP1) ([16] and references therein). A recent study reported that HIPK2 is expressed in the developing pancreatic epithelium from embryonic day e12 to e15 but that the expression becomes preferentially confined to pancreatic endocrine cells at later developmental stages [17]. Moreover, HIPK2 was reportedly able to modulate positively the protein content and transcriptional activity of PDX1, and directly phosphorylate the C-terminal portion of PDX1 [17]. Our data now provide evidence that HIPK2 may be involved in the regulation of PDX1 function in living β-cells through phosphorylation of Ser-269 and regulation of the subnuclear distribution of PDX1. Boucher and colleagues [17] observed an increase of PDX1 transcriptional activity and protein levels upon cotransfection of HEK 293 cells with a plasmid overexpressing HIPK2. By contrast, we were not able in the present studies to detect major changes of PDX1 transcriptional activity upon mutation of serine-269 to a phospho- (glutamic acid) or de-phospho- (alanine) mimetic residue, at least when examined in a heterologous expression system, HEK293 cells (Supplementary Fig. 4). Importantly, in this cellular context, we have not been able to observe effects of glucose on the subnuclear localization of PDX1 (results not shown) suggesting that such changes, as observed in the β-cell, may be the principal route through which changes in Ser269 phosphorylation impact on PDX1 function in the latter context. A further possibility is that HIPK2 is able, as we observed *in vitro*, to phosphorylate PDX1 on more than one site besides serine-269. In fact, the mass spectrometry analysis that we performed on PDX1 overexpressed and immunoprecipitated from clonal β-cells has confirmed that peptide ^211^**SS**G**T**P**S**GGGGGEEPEQDCAV**TS**GEELLAVPPLPPPGGAVPPGVPAAVR^258^ bears at least one phosphate group as earlier observed by Boucher and colleagues [17]. Threonine-214 in this peptide is a likely candidate target for phosphorylation by HIPK2.

In contrast to earlier studies [15] we did not observe differences in stability between wild-type and mutant (Ser-269) PDX1. One significant protocol difference between the present and earlier studies is that we have analyzed more time points, allowing a more precise determination of the half-life. Importantly, the calculated half-life based on our protocol was shorter, at 5 h and 30 min (±4 min), than the previously reported “approximately 8 h” [15]. Also, treatment of β-cells with 100 μg/ml cycloheximide, as undertaken by Humphrey and colleagues, might have affected cellular metabolism and hence protein turnover.

## 5 Conclusion

Our data provide evidence that serine 269 in mouse PDX1 is phosphorylated in living pancreatic β-cells, as revealed through the use of mass spectrometry and confirmed by the generation of a phosphoserine-specific antibody. We also show that this event affects the subnuclear localization of PDX1 in β-cells.
