# Supplementary material for: Pancreatic and duodenal homeobox 1 (PDX1) phosphorylation at serine-269 is HIPK2-dependent and affects PDX1 subnuclear localization
Source: Biochem Biophys Res Commun. 2010 Aug 20;399(2):155–61. doi: 10.1016/j.bbrc.2010.07.035 (PMC2958310; doi:10.1016/j.bbrc.2010.07.035)
Supplement: Supplementary data 1 — Supplementary material. [file mmc1.doc]

**Supplementary data for**

**Pancreatic and duodenal homeobox 1 (PDX1) phosphorylation at serine-269 is**

**HIPK2-dependent and affects PDX1 sub-nuclear localization.**

**^1^To whom correspondence should be addressed:**

**Guy A. Rutter PhD,**

**Professor and Head of Section of Cell Biology**

**Department of Medicine**

**Imperial College London**

**Room 110, Sir Alexander Fleming Building, South Kensington Campus**

**Exhibition road**

**London**

**SW7 2AZ**

**UK**

**Telephone:** +**44 020 7594 3340**

**Secretary's Number: +44 020 7594 3391**

**Fax Number: +44 020 7594 3351**

**e-mail** [**g.rutter@imperial.ac.uk**](../g.rutter@imperial.ac.uk)

**Abbreviations**

CK1(δ), casein kinase 1 (isoform delta); CMV, cytomegalovirus; GSK-3, glycogen synthase kinase 3; JNK, c-Jun NH2-terminal kinase; MAPK, mitogen activated protein kinase; MIN6 β cell, mouse insulinoma pancreatic beta cell; MOI, multiplicity of infection; SAPK, stress activated protein kinase; S269A, a mutation of serine to alanine at position 269; S269E, a mutation of serine to glutamic acid at position 269; siRNA, small interfering RNA.

**MATERIALS AND METHODS**

**Sequencing and mass spectrometric analysis of phosphorylation sites mapping**

Thirty T-75 flasks of INS-1(832/13) cells were infected (MOI 50) with adenovirus expressing *c-myc*-tagged wild-type PDX1­ for 48 h. The cells were then collected and lysed for immunoprecipitation (IP) as described. The IP product was separated by SDS-PAGE and stained with colloidal Commassie blue (SimplyBlue SafeStain, Invitrogen). The gel bands corresponding to PDX1 were excised and all the subsequent mass spectrometry experiments and analysis were performed by “FingerPrints” Proteomic Facility, College of Life Sciences, University of Dundee. Isolated gel bands were processed by in-gel digestion with trypsin. The digested samples were extracted and taken through three stages of analysis using a 4000 QTRAP (Applied Biosystems) mass spectrometer. The stages were as follows: identification of protein(s) by nanospray Liquid Chromatography-MS-MS; precursor ion scanning [Pre 79 or parents of -79 (PO3-)] analysis to detect phosphorylated peptides; data interpretation including *de novo* assignment of phosphorylation sites and final reporting. Ser-269 phosphorylation was further detected using *OrbiTrap* by targeting the site-of-interest containing peptides [1].

***In vitro* phosphorylation by HIPK2, CK1δ and GSK-3β**

GSK-3β was purchased from Upstate (Lake Placid, NY) and CK1δ was prepared as previously described [2].

PDX1 (3 μg) either wild type or S269A mutant were firstly incubated at 37 °C in 20 μl of kinase buffer (10 mM Tris buffered Mops pH 7.5, 20 mM magnesium acetate, 1 mM dithiothreitol, and 0.25 mM ATP) in the presence of the phosphorylating kinase (1.7, 1.5 and 2 pmol for CK1δ, GSK-3β and HIPK2, respectively). After 45 min GSK-3β (1.5 pmol) was added (water in the controls) together with a new mixture of [γ-^33^P] ATP (specific radioactivity 5000 cpm/pmol) and the incubation continued for 15 min. Samples were then blocked with Laemmli buffer and subjected to SDS-PAGE and western blotting. Nitrocellulose membranes were directly exposed to autoradiography and subsequently immunostained with anti-pSer-269 antibodies.

**Electrophoretic mobility shift assay (EMSA)**

DNA-binding properties of wild type, S269A and S269E mutant PDX1 proteins (overexpressed in HEK 293 by adenoviral transduction) were tested by EMSA assay using a mouse insulin I gene promoter A3/A4 element probe that contains a PDX1 binding motif (TAAT). Single stranded DNA oligos sequences were as described in [3] (forward: 5’-CTT ATT AAG ACT ATA ATA ACC CTA AGA CTA-3’; reverse: 5’-TAG TCT TAG GGT TAT TAT AGT CTT AAT AAG-3’). Oligos were annealed, 5’ end labeled with [γ-^32^P] ATP using T4 polynucleotide kinase (Invitrogen), and column-purified (Pharmacia). Nuclear extracts prepared according to [4] from HEK 293 infected with adenoviruses encoding wild-type or mutant forms of PDX1 were incubated with the [^32^P] labeled probe for 30 min. at room temperature in EMSA binding reaction buffer [10 mM Hepes/NaOH pH 7.9, 60 mM KCl, 5 mM MgCl_2_, 5 mM DTT, 4 mM Spermidine, 1 mM EDTA, 1 µg of poly (dI­dC)/poly (dI-dC) and 5% (v/v) glycerol]. One microliter of PDX1 antiserum (kindly provided by Dr. Chris Wright, Vanderbilt University) was added in supershift assays. Samples were run on a 20x20 cm non-denaturing 5% (w/v) polyacrylamide gel (acrylamide/bisacrylamide, 29:1), in 0.5X TBE (50 mM Tris, 50 mM boric acid and 1 mM EDTA) at 120-150 V for 3 to 4 h.

**Transient transfections and luciferase assay**

For luciferase assays, HEK 293 cells seeded in 12-well plate were transfected at 70-90% confluency using Ca^2+^-phosphate. Total DNA (as indicated in different reactions) was mixed with 2.5 M CaCl_2_ and was added drop by drop to an equal volume of 2X Hepes-buffered saline (HBS) in a sterile tube while vortex mixing. Empty expression plasmid was used to maintain a constant total amount of DNA in each well. Cells were harvested 48 h after transfection for western (immuno) blot analyses or for luciferase assays. In the latter case, data are presented as Firefly luciferase activity normalized to *Renilla* *reniformis* luciferase activity (pRL-TK, Promega).

**RESULTS**

**Phosphorylation of PDX1 in intact β-cells: analysis by mass spectrometry***.*

In an effort to identify physiologically relevant phosphorylation sites on PDX1 in living cells, we used adenoviral transduction to overexpress full length PDX1 in clonal INS-1(832/13) β-cells. After immunoprecipitation, we scanned peptides released by trypsin digestion of the protein. Phosphorylation site mapping was then undertaken with a 4000 QTRAP LC/MS/MS system. The sequences generated were searched against those supplied for PDX1 (Mascot Search) and against the National Center for Biotechnology Information (NCBI) non redundant (nr) database (all species).

The PDX1-derived peptide L^262^PSGLSV**pS**PQPSSIAPLRPQEPR^284^, was found to be phosphorylated on Ser-269 (Supplementary Figure 1; in bold, corresponding to S268 both in the human and rat sequence).

Peptide **S**^211^**S**G**T**P**S**GGGGGEEPEQDCAV**TS**GEELLAVPPLPPPGGAVPPGVPAAVR^258^

(Suppl. Fig. 1) was also found to bear at least one phosphate group. However, the precise site of phosphorylation of this peptide was not investigated further.

***In vitro* kinase screening**

In order to identify enzymes able to phosphorylate PDX1 at Ser-269 *in vitro* we preliminarily screened a panel of protein kinases whose expression we confirmed in islets by microarray analysis [5]. We observed that PDX1 can be phosphorylated *in vitro* either by MAPK8 (alias JNK/JNK1/SAPK1), protein kinase Cα (PKCα) [6], and 90 ribosomal S6 kinase (p90Rsk1). Also Aurora A and MAPK11 (alias SAPK2/p38-2/p38Beta, data not shown) phosphorylated PDX1 *in vitro*, even though with less efficiency than JNK, PKCα, and p90Rsk1 (Suppl. Fig. 2).

**Analysis of PDX1 *in vitro* phosphorylation by HIPK2, GSK-3β and CK1δ**

The target consensus sequence for GSK-3 is S/T-X-X-X-pS/pT where pS/pT represents the so called “priming phosphate” i.e. the serine/threonine residue which must be first phosphorylated by another kinase in order for the upstream serine/threonine to be recognized and phosphorylated by GSK-3 [7]. We noted that PDX1 serine residue 269 lying within the sequence S^269^PQPS^273^ could be a good candidate target for phosphorylation by GSK-3 if Ser-273 is already phosphorylated. Also CK1 is a “phosphate-directed” protein kinase [8] and it could in principle phosphorylate Ser-273 if primed by upstream phosphorylation of Ser-269. In order to examine these possible hierarchical phosphorylations of PDX1 promoted by HIPK2, GSK-3 and CK1, we conducted a series of combined *in vitro* phosphorylation trials as reported in Suppl. Fig. 3. CK1δ phosphorylated PDX1 on its own and subsequent addition of GSK-3β caused a highly reproducible increase (71 ± 14 %; *n* = 3) of the overall incorporation of [^33^P] (Suppl. Fig. 3A, left panel). Mutation of Ser-269 to alanine however showed no difference in either phosphorylation by CK1δ or increase of [^33^P] incorporation upon addition of GSK-3β compared to wild-type PDX1 (Suppl. Fig. 3A, right panel); this observation only explains the fact that Ser-269 of PDX1 is not a potential target of either CK1δ or GSK-3β. We further confirmed this result by parallel western blotting with anti-phospho-Ser-269 PDX1 specific antibody: no Ser-269 phosphorylation was detected in either case (Suppl. Fig. 3A). On the other hand, as shown in Fig. 2 and in Suppl. Fig. 3B, HIPK2 alone was able to catalyse the incorporation of phosphate into recombinant PDX1. This was accompanied by increased phosphorylation at Ser-269 as revealed with a phospho-Ser-269 specific PDX1 antibody (Suppl. Fig. 3B).Whilst the incorporation of [^33^P] in the presence of HIPK2 increased further upon subsequent addition of GSK-3β to the reaction, similar changes were seen with wild type and S269A mutant (Suppl. Fig. 3B, left panel), phospho-269-specific antibody could not detect any further increase in signal. These observations suggest that GSK-3β did enhance HIPK2-mediated phosphorylation, but at a site distinct from Ser-269 (Suppl. Fig. 3B).

We deduced similar conclusions also from experiments run with synthetic peptides (data not shown). We performed a phosphorylation analysis of the synthetic peptide C^263^SGLSV**S^269^**PQP**S^273^**SIAPLRPQEP^283^ reproducing the sequence 263-283 of mouse PDX1 either as such and also bearing a phosphogroup on serine residue 269: C^263^SGLSV**pS^269^**PQP**S^273^**SIAPLRPQEP^283^. We showed that CK1δ phosphorylates well the peptide when serine residue 269 has been previously modified with a phosphate, while it is unable to phosphorylate the unmodified peptide, as it would be expected from CK1 consensus target sequence: pS/pT-Xn-S/T (with n = 2 much more effective than n = 1 or n = 3) [8;9]. Therefore we hypothesize that CK1δ phosphorylates the serine residue in position 273, this will require further experiments to be surely confirmed. Moreover we observed that GSK-3β, while able to highly efficiently phosphorylate a peptide containing focal adhesion kinase (FAK) residues 714-730, was completely unable to phosphorylate either the phospho-S269-peptide and the unmodified peptide, suggesting that GSK-3 might target other PDX1 residues rather than Ser-269 or Ser-273 as reported by Humprey and colleagues [10]. Further studies will be needed to examine this question.

**Phosphorylation on Ser-269 does not affect the DNA-binding and transcriptional activity of PDX1**

Because the C-terminal domain of PDX1 supposedly play an important role in controlling transcriptional activity [11] we sorted to examine whether the dephospho- or phospho-mimetic mutants of PDX1 (S269A and S269E) displayed a deviation in transcriptional activity from the wild type protein. Primarily we explored the impact of Ser-269 phosphorylation on DNA-binding activity of PDX1, for which a 30 basepair fragment of the mouse I insulin promoter, containing a PDX1 binding motif (TAAT) [12-14], was used in electrophoretic mobility shift assays (EMSA) (Suppl. Fig. 4A). For this purpose recombinant PDX1 protein was overexpressed and immunoprecipitated from HEK 293 cells since these cells do not express endogenous PDX1. As shown before [15], nuclear lysate from cells over-expressing wild-type PDX1 showed strong binding to the radiolabeled probe derived from this promoter element (Suppl. Fig. 4A). The binding was specific and was blocked efficiently by competition with unlabeled probe. Furthermore bound signal was super-shifted by anti-PDX1 antibody. When similar assays were carried out with nuclear lysates expressing mutant PDX1 (S269A and S269E), the strength of binding and super-shift were indistinguishable from wild-type protein. Thus, neither phosphomimetic nor dephospho-mimetic mutation of PDX1 at Ser-269 had any effect on DNA-binding activity of PDX1.

We next explored if Ser-269 phosphorylation of PDX1 influenced its transactivation of the preproinsulin promoter, using a luciferase promoter-reporter system in a heterologous cell line. Transfection of HEK 293 cells with equivalent amounts of plasmids encoding *c*-*myc*-tagged wild-type, S269A or S269E PDX1 led to the same degree of induction of the human preproinsulin promoter (normalized to CMV promoter as monitored by the expression of *Renilla reniformis* luciferase), compared to cells transfected with empty vector only (Suppl. Fig. 4B). Transfection efficiency in these experiments was verified by western blotting using anti-*c*-*myc* antibody and the levels of all three different forms of PDX1 were similar (Suppl. Fig. 4C). These data indicated that dephospho- (to alanine) or phospho- (to glutamic acid) mimetic mutation of Ser-269 did not alter the stability of the protein.

**SUPPLEMENTARY FIGURE LEGENDS**

**Supplementary Figure 1.**

**Phosphorylation site mapping of PDX1 by mass spectrometry**

Automated nLC-MS/MS analysis using Precursor Ion Scan of *m/z* –79 on tryptic digests of *c-myc*-PDX1 immunoprecipitated from adenovirally-infected INS-1 (832/13) cells. Mass and charge states for the precursor ions detected over the nLC separation is shown. Peaks corresponding to peptides ions which were subsequently identified as *c-myc*-PDX1 derived phosphorylated peptides are indicated. Peptide sequence is shown with phosphorylated residue in bold. Peptide E^259^GLLPSGLSV**pS^269^**PQPSSIAPLRPQEPR is present with two peaks, one at m/z 930.5 corresponding to the deprotonated peptide ion charge state (M-3H) and the other at *m/z* 1395.8 corresponding to charge state (M-2H). This peptide was found phosphorylated on the indicated Serine residue 269 (in bold).

Peptide **S^211^S**G**T**P**S**GGGGGEEPEQDCAV**TS**GEELLAVPPLPPPGGAVPPGVPAAVR was also found to be phosphorylated on a Serine or Threonine residue.

*m/z*, mass to charge ratio in atomic units (amu); cps, counts per second.

**Supplementary Figure 2.**

**Kinase screen for PDX-1 phosphorylation *in vitro***

About 1 µg of PDX-1 wild type was used in the assays. All kinases were active recombinant enzymes, either from commercial suppliers or from the University of Dundee. Each kinase was used at 50 ng per reaction. The kinase buffer for PKCα was 10 mM Hepes pH 7.4, 10 mM β-mercaptoethanol, 0.5 mM CaCl_2_, 0.25 mg/ml phosphatidyl serine, 0.05 mg/ml diacylglycerol, 0.01% Triton X-100.

All other kinases were used in 20 mM Tris pH 7.5, 10 mM β-mercaptoethanol. The kinase assays were carried out in duplicate at 30^o^C in the presence of 10 mM Mg-acetate and 0.1 mM [γ-^32^P]ATP. The [γ-^32^P]ATP was freshly prepared for every set of experiments. The reactions were stopped by adding SDS-loading buffer and then analysed by SDS-PAGE, colloidal Coomassie staining and autoradiography. The reaction buffers for the kinetic study were supplemented with BSA in order to stabilise the kinases. The only exception was PKC, because PKC is inactivated by BSA. The kinase screen was performed by Kinasource Limited, James Black Centre, Dundee

**Supplementary Figure 3.**

**PDX1 *in vitro* phosphorylation by CK1δ, HIPK2 and GSK-3β.**

Recombinant PDX1 (3 μg), either wild type (WT) or S269A mutant, was firstly incubated at 37 °C in 20 μl of kinase buffer (10 mM Tris buffered Mops pH 7.5, 20 mM magnesium acetate, 1 mM dithiothreitol, 250 µM ATP) in the presence of the phosphorylating kinases (A) CK1δ (1.7 pmol), or (B) HIPK2 (2 pmol) and GSK-3β (1.5 pmol). After 45 min, GSK-3β (1.5 pmol) was added (water was used in the controls) together with a new mixture of [γ-^33^P] ATP (specific activity 5000 cpm/pmol) and the incubation continued for 15 min. Samples were then blocked with Laemmli buffer and subjected to SDSPAGE and western blotting. Nitrocellulose membranes were directly exposed to autoradiography and subsequently immunostained with anti-phospho-Ser-269 antibodies.

**Supplementary Figure 4.**

**Effect of Ser-269 phosphorylation on PDX1 DNA-binding and transcriptional activities**

(A) The indicated amount of HEK 293 cell nuclear extracts over-expressing wild-type (WT) or mutant forms (S269A, S269E) of PDX1 were incubated with [γ-^32^P] labeled oligonucleotide corresponding to the mouse insulin I gene promoter (A3/A4 element) as indicated, with or without 1 μl of PDX1 antibody for supershift assay, or with 5x, 10x or 20x un-labeled oligonucleotides as binding competitor. The binding reaction mixture was migrated on a 20 x 20 cm non-denaturing 5% (w/v) polyacrylamide gel (acrylamide/bisacrylamide, 20:1). The gel was dried and subjected to autoradiography. (B, C) HEK 293 cells were transfected using Ca^2+^-phosphate with 0.1 μg of pcDNA3 empty vector or pcDNA3-PDX1 wild type (WT) or serine to alanine (S269A) or serine to glutamic acid (S269E) mutants, 0.1 μg *PPI* promoter luciferase, and 0.01 μg *Renilla-*luciferase*.* (B) Cells were harvested 48 h following transfection and protein cell extraction for luciferase assay or western blot analyses were carried out. (C) Equal amount of whole cell lysates from the luciferase assay were separated by SDS-PAGE and analysed by western blot using anti-*c-myc* antibody. The relative PDX1 expression level is shown graphically. **p<0.01, the experiment was performed in triplicate. Mean values from three independent experiments are shown.

**REFERENCES**

[1] M. Scigelova, A. Makarov, Orbitrap mass analyzer--overview and applications in proteomics. Proteomics. 6 Suppl 2 (2006) 16-21.

[2] G. Cozza, A. Gianoncelli, M. Montopoli, L. Caparrotta, A. Venerando, F. Meggio, L.A. Pinna, G. Zagotto, S. Moro, Identification of novel protein kinase CK1 delta (CK1delta) inhibitors through structure-based virtual screening. Bioorg.Med.Chem.Lett. 18 (2008) 5672-5675.

[3] S.K. Chakrabarti, J.C. James, R.G. Mirmira, Quantitative assessment of gene targeting in vitro and in vivo by the pancreatic transcription factor, Pdx1. Importance of chromatin structure in directing promoter binding. J.Biol.Chem. 277 (2002) 13286-13293.

[4] E. Schreiber, P. Matthias, M.M. Muller, W. Schaffner, Rapid detection of octamer binding proteins with 'mini-extracts', prepared from a small number of cells. Nucleic Acids Res. 17 (1989) 6419.

[5] L.E. Parton, P.J. McMillen, Y. Shen, E. Docherty, E. Sharpe, F. Diraison, C.P. Briscoe, G.A. Rutter, Limited role for SREBP-1c in defective glucose-induced insulin secretion from Zucker diabetic fatty rat islets: a functional and gene profiling analysis. Am.J.Physiol Endocrinol.Metab 291 (2006) E982-E994.

[6] Y.M. Tian, V. Urquidi, S.J. Ashcroft, Protein kinase C in beta-cells: expression of multiple isoforms and involvement in cholinergic stimulation of insulin secretion. Mol.Cell Endocrinol. 119 (1996) 185-193.

[7] P. Cohen, S. Frame, The renaissance of GSK3. Nat.Rev.Mol.Cell Biol. 2 (2001) 769-776.

[8] H. Flotow, P.R. Graves, A.Q. Wang, C.J. Fiol, R.W. Roeske, P.J. Roach, Phosphate groups as substrate determinants for casein kinase I action. Journal of Biological Chemistry 265 (1990) 14264-14269.

[9] J.W. Perich, F. Meggio, E.C. Reynolds, O. Marin, L.A. Pinna, Role of phosphorylated aminoacyl residues in generating atypical consensus sequences which are recognized by casein kinase-2 but not by casein kinase-1. Biochemistry 31 (1992) 5893-5897.

[10] R.K. Humphrey, S.M. Yu, L.E. Flores, U.S. Jhala, Glucose regulates steady-state levels of PDX1 via the reciprocal actions of GSK3 and Akt kinases. J.Biol.Chem. 285 (2009) 3406-3416.

[11] A. Liu, B.M. Desai, D.A. Stoffers, Identification of PCIF1, a POZ domain protein that inhibits PDX-1 (MODY4) transcriptional activity. Mol.Cell Biol. 24 (2004) 4372-4383.

[12] H. Ohlsson, K. Karlsson, T. Edlund, IPF1, a homeodomain-containing transactivator of the insulin gene. EMBO J. 12 (1993) 4251-4259.

[13] J. Jonsson, L. Carlsson, T. Edlund, H. Edlund, Insulin-promoter-factor 1 is required for pancreas development in mice. Nature 371 (1994) 606-609.

[14] M. German, S. Ashcroft, K. Docherty, H. Edlund, T. Edlund, S. Goodison, H. Imura, G. Kennedy, O. Madsen, D. Melloul, ., The insulin gene promoter. A simplified nomenclature. Diabetes 44 (1995) 1002-1004.

[15] D. Melloul, Y. Ben-Neriah, E. Cerasi, Glucose modulates the binding of an islet-specific factor to a conserved sequence within the rat I and the human insulin promoters. Proc.Natl.Acad.Sci.U.S.A 90 (1993) 3865-3869.
