# Supplementary material for: Pancreatic and duodenal homeobox 1 (PDX1) phosphorylation at serine-269 is HIPK2-dependent and affects PDX1 subnuclear localization
Source: Biochem Biophys Res Commun. 2010 Aug 20;399(2):155–61. doi: 10.1016/j.bbrc.2010.07.035 (PMC2958310; doi:10.1016/j.bbrc.2010.07.035)
Supplement: Supplementary figures 2 — Supplementary figures. [file mmc2.ppt]

## Slide 1
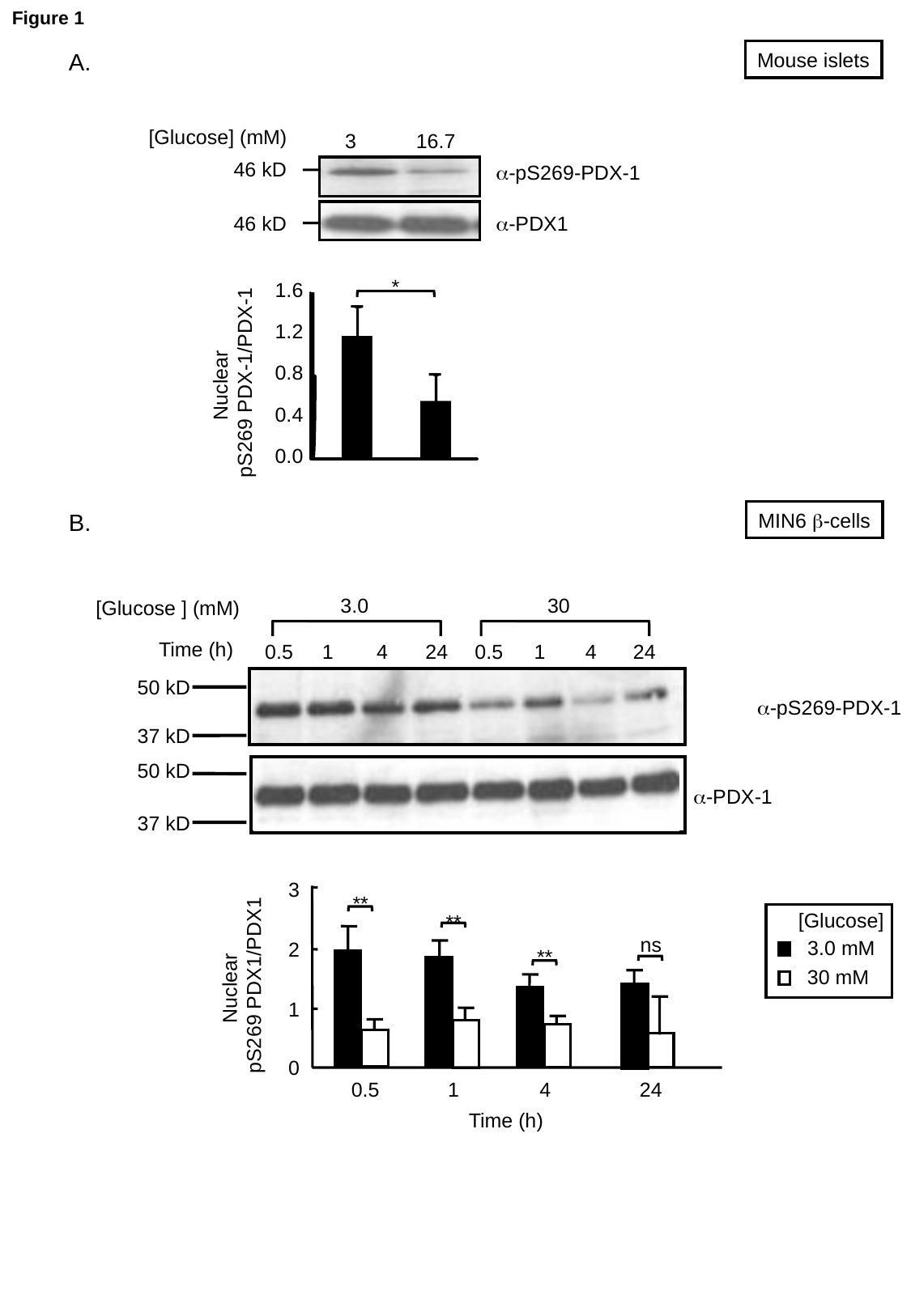

Figure 1
A.
Mouse islets
[Glucose] (mM)
3
16.7
46 kD
a-pS269-PDX-1
a-PDX1
46 kD
*
1.6
1.2
Nuclear
pS269 PDX-1/PDX-1
0.8
0.4
0.0
B.
MIN6 b-cells
3.0
30
[Glucose ] (mM)
Time (h)
0.5
1
24
0.5
1
24
4
4
50 kD
37 kD
50 kD
a-PDX-1
37 kD
a-pS269-PDX-1
3
**
**
[Glucose]
ns
3.0 mM
2
**
Nuclear
pS269 PDX1/PDX1
30 mM
1
0
0.5
1
4
24
Time (h)

## Slide 2
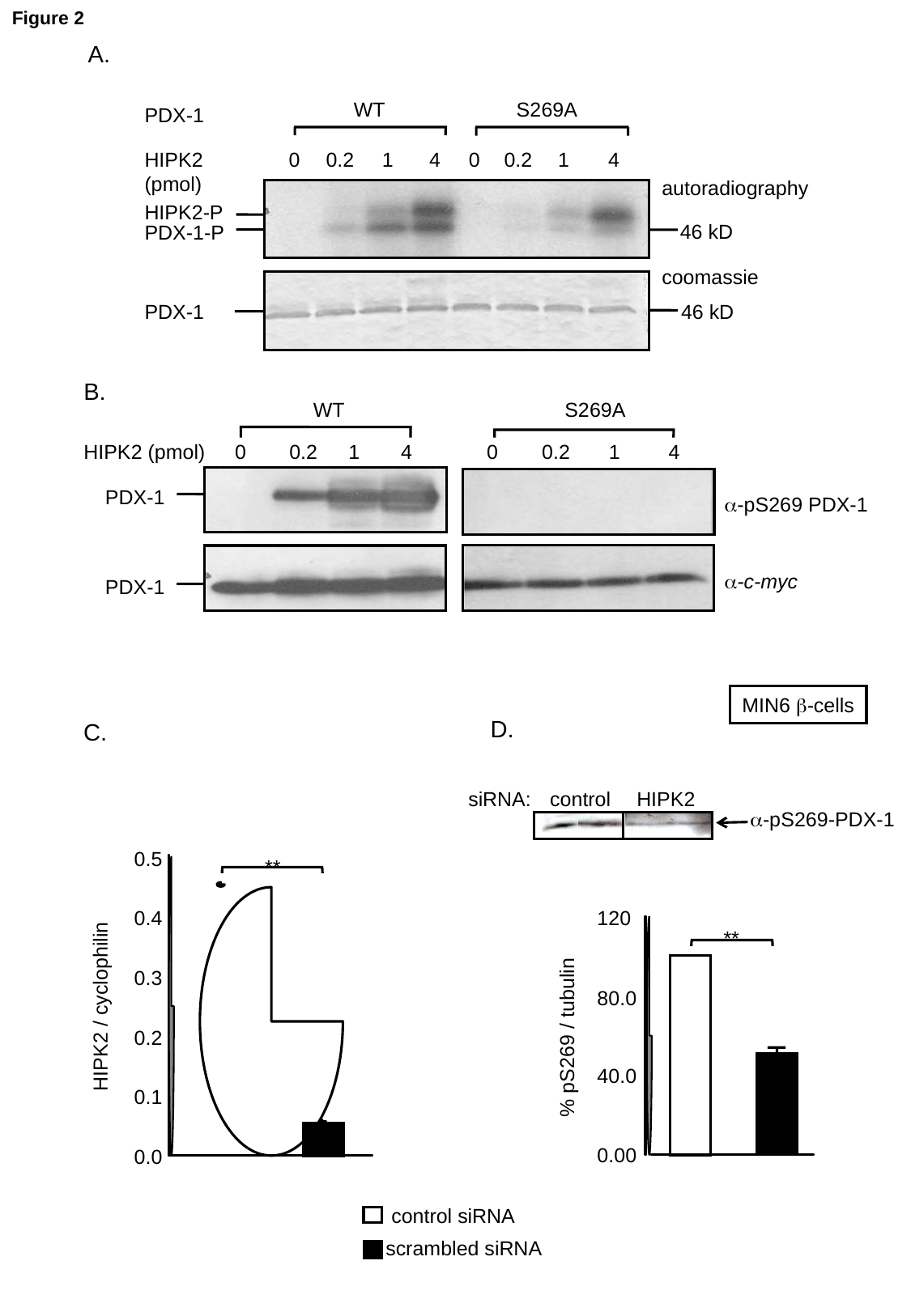

Figure 2
A.
WT
S269A
PDX-1
HIPK2 (pmol)
0
0.2
1
4
0
0.2
1
4
autoradiography
HIPK2-P
46 kD
PDX-1-P
coomassie
PDX-1
46 kD
B.
WT
S269A
HIPK2 (pmol)
0
0.2
1
4
0
0.2
1
4
PDX-1
a-pS269 PDX-1
a-c-myc
PDX-1
MIN6 b-cells
D.
C.
siRNA:
control
HIPK2
a-pS269-PDX-1
0.5
**
0.4
0.3
HIPK2 / cyclophilin
0.2
0.1
0.0
120
**
80.0
% pS269 / tubulin
40.0
0.00
 control siRNA
scrambled siRNA

## Slide 3
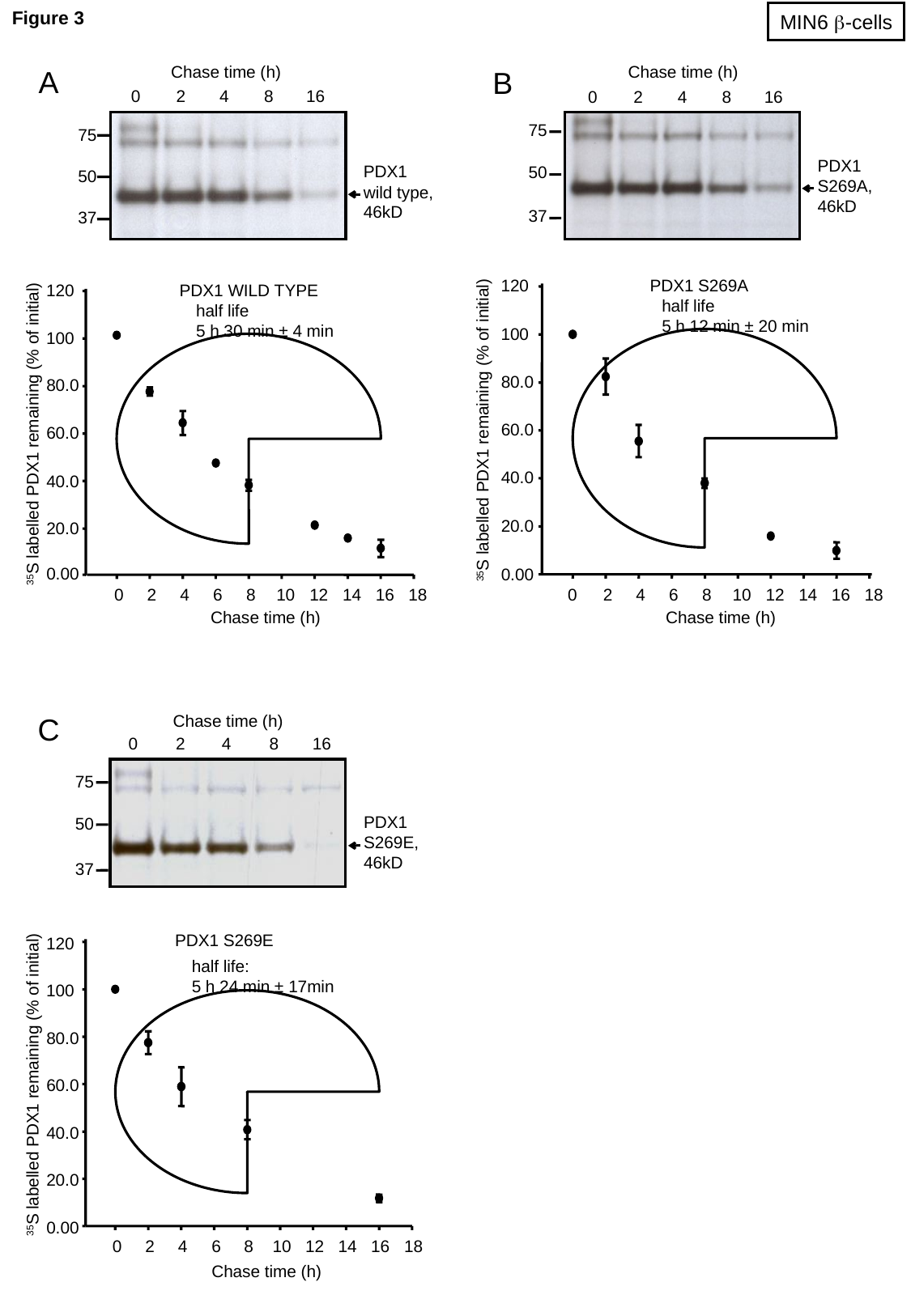

Figure 3
MIN6 b-cells
Chase time (h)
Chase time (h)
0
2
4
8
16
75
50
37
PDX1
wild type,
46kD
A
B
0
2
4
8
16
75
50
37
PDX1
S269A,
46kD
PDX1 S269A
120
100
80.0
60.0
40.0
20.0
0.00
PDX1 WILD TYPE
120
100
80.0
60.0
40.0
20.0
0.00
half life
5 h 12 min ± 20 min
half life
5 h 30 min ± 4 min
35S labelled PDX1 remaining (% of initial)
35S labelled PDX1 remaining (% of initial)
0
2
4
6
8
10
12
14
16
18
0
2
4
6
8
10
12
14
16
18
Chase time (h)
Chase time (h)
Chase time (h)
0
2
4
8
16
75
50
37
PDX1
S269E,
46kD
C
PDX1 S269E
120
100
80.0
60.0
40.0
20.0
0.00
half life:
5 h 24 min ± 17min
35S labelled PDX1 remaining (% of initial)
0
2
4
6
8
10
12
14
16
18
Chase time (h)

## Slide 4
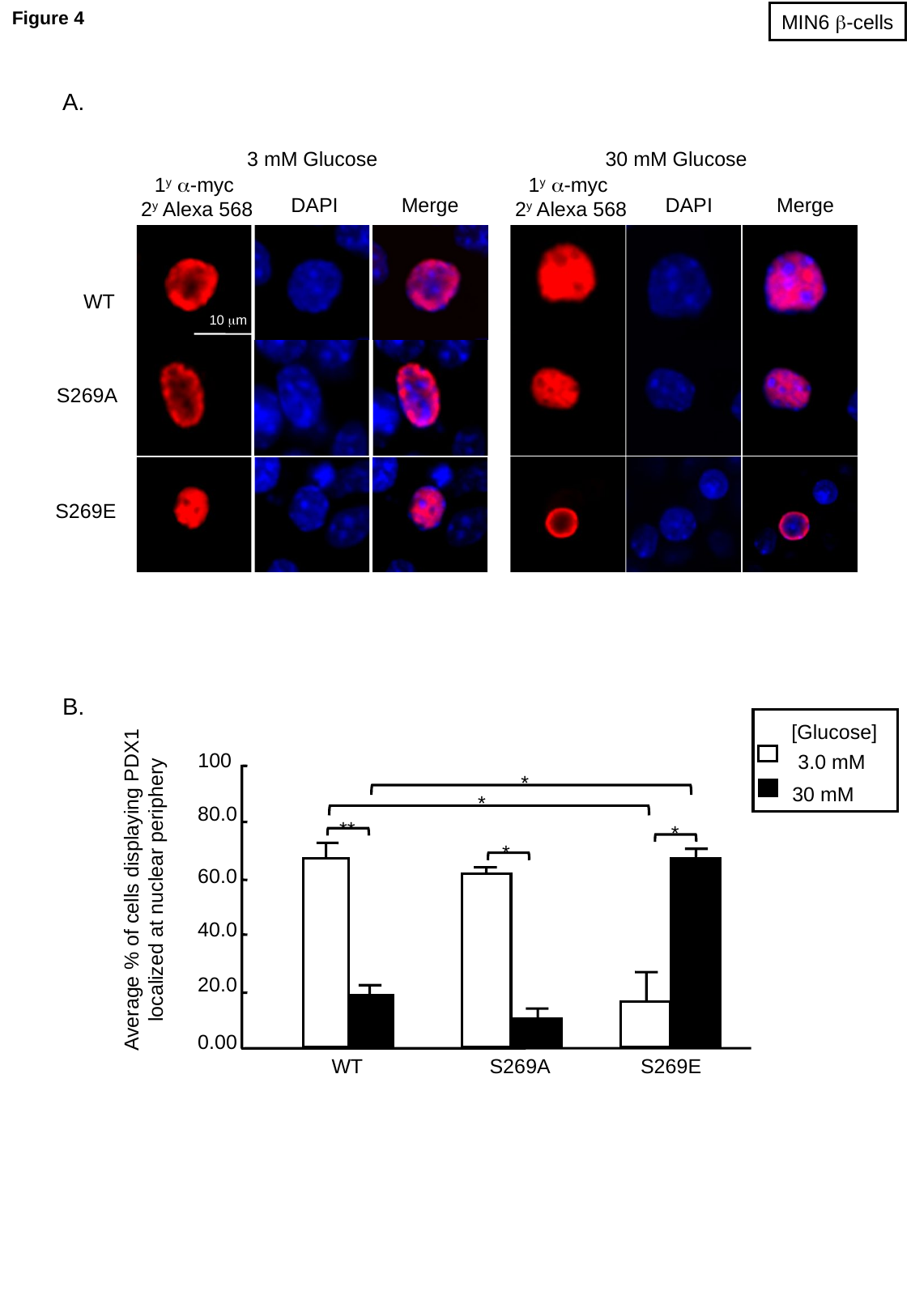

Figure 4
MIN6 b-cells
A.
3 mM Glucose
30 mM Glucose
1y a-myc
2y Alexa 568
1y a-myc
2y Alexa 568
DAPI
Merge
DAPI
Merge
WT
10 mm
S269A
S269E
B.
[Glucose]
 3.0 mM
30 mM
100
*
*
80.0
**
*
*
60.0
Average % of cells displaying PDX1 localized at nuclear periphery
40.0
20.0
0.00
WT
S269A
S269E
